# Opportunistic digestive parasitic infections in adults infected with HIV: epidemiological expression

**DOI:** 10.1186/1471-2334-14-S2-P39

**Published:** 2014-05-23

**Authors:** L Badaoui, G Dabo, R Bensghir, I Halim, M Soussi Abdallaoui, A Chakibi, K El Filahi Marhoum

**Affiliations:** 1 Ibn Rochd University Hospital, Casablanca, Morocco

## Introduction

In Morocco as in many African countries, AIDS and its procession of opportunistic infections are a major cause of morbidity and mortality. The purpose of study was to determine the frequency of digestive opportunistic parasitosis in patients infected with HIV.

## Materials and methods

A retroprospective study conducted 20 months in the department of infectious diseases. Were included all patients infected with HIV and opportunistic intestinal parasitosis confirmed in EPS. Data were collected on computer files (Nadis) and analyzed on Epi Info.

## Results

Among the 70 patients involved, the average age was 37 years with a slight male predominance. The median CD4 was 62cel/mm ³. Digestive opportunistic parasitic were indicative of HIV infection in 54 cases (77%) and in 16 cases (23%) they occurred at the waning of treatment failure. All patients had diarrhea. These opportunistic parasitic agents were isolated only in 56 cases: the cryptosporidiosis (40%), microsporidia (31%), Isospora belli (6%) and Cyclospora (3%). They were associated in 14 cases including Cryptosporidiosis+ Microsporidiosis (18.57%) and Cryptosporidiosis+ Isosporiasis (1.43%) in HARRT the outcome was favorable for 86% of patients (n = 60) and mortality was 14% (n=10).

## Conclusion

Opportunistic digestive parasites remain common in our context because of late diagnosis of HIV / AIDS. Early detection of HIV will prevent them.

